# Statistical shape modelling of hip and lumbar spine morphology and their relationship in the MRC National Survey of Health and Development

**DOI:** 10.1111/joa.12631

**Published:** 2017-05-31

**Authors:** Anastasia V. Pavlova, Fiona R. Saunders, Stella G. Muthuri, Jennifer S. Gregory, Rebecca J. Barr, Kathryn R. Martin, Rebecca J. Hardy, Rachel Cooper, Judith E. Adams, Diana Kuh, Richard M. Aspden

**Affiliations:** ^1^ Aberdeen Centre for Arthritis and Musculoskeletal Health School of Medicine Medical Sciences and Nutrition University of Aberdeen Aberdeen UK; ^2^ MRC Unit for Lifelong Health and Ageing at UCL London UK; ^3^ Manchester Academic Health Science Centre Manchester Royal Infirmary Central Manchester University Hospitals NHS Foundation Trust Manchester UK; ^4^ Present address: Medicines Monitoring Unit (MEMO) Division of Molecular & Clinical Medicine School of Medicine Ninewells Hospital & Medical School University of Dundee Mailbox 2, Level 7 Dundee DD1 9SY UK

**Keywords:** ageing, hip, lumbar spine, morphology, statistical shape modelling

## Abstract

The anatomical shape of bones and joints is important for their proper function but quantifying this, and detecting pathological variations, is difficult to do. Numerical descriptions would also enable correlations between joint shapes to be explored. Statistical shape modelling (SSM) is a method of image analysis employing pattern recognition statistics to describe and quantify such shapes from images; it uses principal components analysis to generate modes of variation describing each image in terms of a set of numerical scores after removing global size variation. We used SSM to quantify the shapes of the hip and the lumbar spine in dual‐energy x‐ray absorptiometry (DXA) images from 1511 individuals in the MRC National Survey of Health and Development at ages 60–64 years. We compared shapes of both joints in men and women and hypothesised that hip and spine shape would be strongly correlated. We also investigated associations with height, weight, body mass index (BMI) and local (hip or lumber spine) bone mineral density. In the hip, all except one of the first 10 modes differed between men and women. Men had a wider femoral neck, smaller neck‐shaft angle, increased presence of osteophytes and a loss of the femoral head/neck curvature compared with women. Women presented with a flattening of the femoral head and greater acetabular coverage of the femoral head. Greater weight was associated with a shorter, wider femoral neck and larger greater and lesser trochanters. Taller height was accompanied by a flattening of the curve between superior head and neck and a larger lesser trochanter. Four of the first eight modes describing lumbar spine shape differed between men and women. Women tended to have a more lordotic spine than men with relatively smaller but caudally increasing anterior‐posterior (a‐p) vertebral diameters. Men were more likely to have a straighter spine with larger vertebral a‐p diameters relative to vertebral height than women, increasing cranially. A weak correlation was found between body weight and a‐p vertebral diameter. No correlations were found between shape modes and height in men, whereas in women there was a weak positive correlation between height and evenness of spinal curvature. Linear relationships between hip and spine shapes were weak and inconsistent in both sexes, thereby offering little support for our hypothesis.

In conclusion, men and women entering their seventh decade have small but statistically significant differences in the shapes of their hips and their spines. Associations with height, weight, BMI and BMD are small and correspond to subtle variations whose anatomical significance is not yet clear. Correlations between hip and spine shapes are small.

## Introduction

The anatomical shape of bones and joints is crucial to their function, yet difficult to quantify. In diarthrodial joints, smooth articulation maximises the range of motion while minimising the risk of regions of high stress that might damage the cartilage or other component tissues. Consequently, malformations of joint shapes, such as femoroacetabular impingement in the hip, leading to stress concentrations, is increasingly recognised as a risk factor for joint degeneration and osteoarthritis (Khan et al. 2016). Other morphological features, such as a longer, thinner femoral neck, may predispose to increased risk of fractured neck of femur (Beck et al. 2000). The lumbar lordosis in the spine is an adaptation suggested to be essential for an habitual upright stance in *homo sapiens* and a key factor in the ability of humans to carry up to three times their own bodyweight, far in excess of other primates (Farfan, 1978). Despite the importance of joint shape to normal function and disease, studies of the natural morphologies of joints, how they change with age and how the shapes of different joints might be inter‐related are still relatively uncommon. Joints are often considered in isolation, yet the concept of a kinetic chain in which forces are transmitted through a series of joints has been common in movement studies for many years (Steindler, 1955).

Describing the shape of an object as complicated as a joint is difficult. Statistical shape modelling (SSM) is a method of image analysis employing pattern recognition statistics to describe and quantify such shapes from images, mostly in two dimensions but increasingly in 3D (Neogi et al. 2013; Barr et al. 2016). This method has been used to describe and quantify the variations in the shape of complex anatomical structures, such as the knee, hip and spine (Smyth et al. 1999; Gregory et al. 2004; Meakin et al. 2008; Barr et al. 2009), using a wide range of imaging modalities including radiography (Barr et al. 2012; Nelson et al. 2016a), dual energy X‐ray absorptiometry (DXA) (Goodyear et al. 2013), CT/pQCT (Bredbenner et al. 2014; Varzi et al. 2015) and magnetic resonance imaging (MRI; Meakin et al. 2009; Neogi et al. 2013; Pavlova et al. 2014). The power of SSM lies in its ability to quantify variations in morphology and it has recently shown promise as a biomarker for a number of musculoskeletal disorders.

In the spine, SSM has proved useful for identifying existing vertebral fractures, often missed during routine reporting (Smyth et al. 1999; Roberts et al. 2007, 2012). There is increasing awareness that simply describing the spine by its lordosis or kyphosis angle is not sufficient to describe the morphology accurately (Roussouly et al. 2005; Been & Kalichman, 2014) and that important information relating to subtle variations in curvature can be missed (Meakin et al. 2009; Ali et al. 2012). SSM of the lumbar spine has shown that each individual has a characteristic shape that is maintained during different postures and has been linked to lifting techniques with implications for management and prevention of back pain (Meakin et al. 2009; Pavlova et al. 2014). Familial correlations have been observed, indicating a genetic component to spine shape (Dryden et al. 2008).

In the hip, SSM has been used for predicting osteoporotic (OP) fracture of the neck of femur and ROC curves suggest it is comparable to bone mineral density in this regard (Gregory et al. 2004; Baker‐Lepain et al. 2011; Whitmarsh et al. 2012; Goodyear et al. 2013). Detecting early osteoarthritis (OA) and monitoring its progression is essential for trials of new therapeutic agents but sensitive biomarkers of disease are proving elusive. SSM scores have shown strong associations with radiographic osteoarthritis (OA) of the hip, including total hip replacement (THR; Gregory et al. 2007; Lynch et al. 2009; Waarsing et al. 2010; Barr et al. 2012; Nelson et al. 2014) and the knee (Bredbenner et al. 2010; Neogi et al. 2013). A number of genetic markers have been associated either with hip shape directly, or as modifiers of the link between hip shape and development of OA (Waarsing et al. 2011; Baker‐Lepain et al. 2012; Lindner et al. 2015). SSM has also proved useful for studying bone loss due to spinal cord injury (Varzi et al. 2015), and disorders of the foot and ankle (Milliken et al. 2014; Nelson et al. 2016b).

Each of these studies has investigated the link between the shape of a single region of the skeleton and clinical or biomechanical outcomes. All of these joints, however, are connected in the kinetic chain of the weight‐bearing skeleton. Whereas links between different regions have been observed in disorders such as osteoarthritis (for example between hip shape and knee osteoarthritis in the study of osteoporotic fractures and the Johnston County studies; Wise et al. 2014; Nelson et al. 2016a), and knee and lumbar spine in the Chingford study (Hassett et al. 2006), no studies have yet created shape models from different regions in the same people.

Many studies of joint morphology are limited by their sample number (*n* = 9–800) (Cil et al. 2005; Bailey et al. 2016), geometrical (Masharawi et al. 2012; Shefi et al. 2013) or external measurement methods (Wojtys et al. 2000) and often lack heterogeneity in terms of health and disease state in their cohorts. Here we use data from the Medical Research Council (MRC) National Survey of Health and Development (NSHD), the oldest of the British birth cohort studies, to investigate the variations in hip and spine shapes in over 1500 individuals approaching early old age. We compare men and women and explore associations between hip and spine shapes, which we hypothesise will be strongly related, and how these may be related to body habitus and bone mineral density. These data provide a foundation for future studies of relationships between factors across life and joint shapes.

## Materials and methods

### Study cohort

The NSHD is a British birth cohort study of 5362 individuals born in the same week in March 1946 in England, Scotland and Wales, who have been followed‐up over 20 times since birth (Kuh et al. 2011; Stafford et al. 2013). Between ages 60 and 64 years, study members still alive and with a known current address in mainland Britain were invited for assessment, including DXA imaging, at one of six clinical research facilities (CRFs) [Birmingham, Cardiff, Edinburgh, Manchester, London (Guys St Thomas' and UCLH)]; those unable or unwilling to attend a CRF were offered a home visit by a research nurse. Those who participated at age 60–64 have been described in detail elsewhere and shown to be largely representative of the whole cohort and of individuals in the general population born in mainland Britain at that time (Stafford et al. 2013). Weight and height were measured according to standard protocols and body mass index (BMI) was calculated from weight /(height)^2^. Ethical approval for the study was obtained from the Central Manchester Research Ethics Committee (07/H1008/245) and the Scottish A Research Ethics Committee (08/MRE00/12). Written informed consent was obtained from each participant.

### Hip and spine DXA images

Images were obtained using Hologic QDR 4500 Discovery DXA scanners (Kuh et al. 2011) at the six CRF centres. Five centres had scanners with rotating C‐arms, allowing individuals to lie supine for the entire scanning process, and one used a scanner with a fixed C‐arm, requiring patients to be moved between scans so the spine was imaged with the individual in lateral decubitus. All hip images were acquired with feet placed at 15° of internal rotation. Only right hip images were used for analysis and were supplied as anonymised raw files. Spine images were supplied to Aberdeen as anonymised DICOM files. All images were converted to 8‐bit bitmap images using matlab and image j 1.47v (NIH, USA). To aid visualisation, a bandpass fast Fourier transform filter, suppressing horizontal stripes, was applied to all spine images using imagej 1.47v (NIH, USA) to smooth breathing artefact lines often seen in the thoracic region. Images were reflected about a vertical axis to enable visual consistency with previous spine shape models (Meakin et al. 2009). Hip images were unmodified. As well as the images, bone outcomes used in this analysis were areal Bone Mineral Density (BMD) measured from total hip and lumbar spine using standard protocols on each scanner.

### Statistical shape modelling

SSM is a statistical method used to identify and quantify variations in the shape of an object described by a set of landmark points; it has been described in detail previously (Cootes et al. 1994; Gregory et al. 2004; Meakin et al. 2009). Briefly, a series of points were placed around the area of interest in each image. The point coordinates underwent Procrustes transformation to scale, rotate and translate the points to lie on the same scale, thereby removing influences of overall size. Principal components analysis was then performed to derive orthogonal modes describing variations in shape within the sample. Raw mode scores were normalised by the sample standard deviation resulting in a set of mode scores for each image, in units of standard deviations, describing how much that image varied from the mean shape (score = 0 for each mode) for the whole cohort. Scree plots of the percentage variance described by each mode were used to determine how many modes to include for each model.

**Figure 1:**
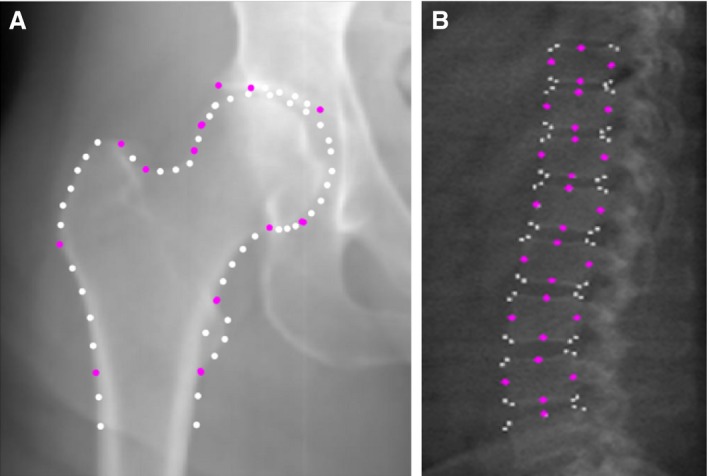
Point placements for the hip and the spine statistical shape models.

Custom‐made ssm software (Shape, Aberdeen University) was used to create a template of points to describe the shape of each joint. The hip template consisted of 68 points describing the proximal femur, associated osteophytes where present, and acetabulum (Fig. 1A). The 89‐point spine template was built to include all vertebrae that were consistently visible in all images, extending from the fifth lumbar vertebra (L5) to the 10th thoracic vertebra (T10) (Fig. 1B). Following an automatic search, each of the images was checked and points manually adjusted where necessary. The SSM was built with both males and females together.

### Repeatability

A set of 50 images were selected at random from the dataset, ensuring that each CRF was equally represented. Intra‐ and inter‐rater repeatability of manual point placement was assessed. Both sets of images were independently marked by two observers (AVP, FRS) and one observer marked the hip (FRS) or spine (AVP) images twice. Repeatability was measured as the difference, in pixels, between coordinates of corresponding points. The mean intra‐ and inter‐rater repeatability was 2.2 and 2.6 pixels, respectively, for the hip and 1.4 and 2.2 pixels, respectively, for the spine. These errors are small considering that the average image size in pixels was 300 × 252 for the hips and 1200 × 400 for the spines, with a typical vertebra measuring approximately 80 × 60 pixels.

### Statistical analysis

Histograms and normal Q‐Q plots were visually inspected and used to determine normality of each joint shape mode score. Because we expected differences in mode scores between sexes, sex‐specific means and standard deviations (SDs) for each joint shape mode were estimated and sex differences were formally tested using Student's *t*‐tests. We used Pearson's correlation (*r*) to assess whether there were linear relationships between hip and spine morphologies in men and women separately. Partial correlations adjusting for CRF were used to test whether there were linear relationships between each joint shape mode and height, weight, BMI and local BMD, i.e. total hip BMD for hip mode scores and lumbar spine BMD for spine mode scores. We used *r* > 0.1 as a threshold to aid interpretation. *P*‐values are not given because in a sample this large, even small correlations (i.e. *r* < 0.1) are statistically significant at *P *< 0.05, making it difficult to make meaningful interpretations. All analyses were conducted using stata version 14.0 (StataCorp, College Station, TX, USA).

## Results

### Participant demographics

Of the 2856 study participants invited to attend the CRF, 2229 responded and 1690 attended a CRF (the rest having a home visit). DXA scans were performed on 1656 participants, with 1636 having their hips imaged, 1601 having their spine imaged and 1581 having scans of both joints. Three hip images were excluded due to extreme internal rotation of the joint, shown by foreshortening of the femoral neck, leaving a final number of 1633 hip images which were used to build the hip SSM. From the spine images, 72 were excluded: 41 due to being unable to clearly determine all vertebral outlines, 23 because of scanning artefacts, five had incomplete images, metalwork in two and excessive axial rotation in one, leaving 1529 spine images which were used to build the spine SSM. Mode scores from 1511 participants with good quality scans of both the hip and spine were analysed in this study. Those with images excluded were more likely to be female (46/70 individuals, 65.7%; *P = *0.022) and have a higher mean bodyweight than that of the included cohort [87.5 (23.0) kg, vs. 78.1 (14.3) kg, *P *< 0.001, respectively]. Hence their mean BMI was just inside the obese category: 30.9 (8.0) kg m^−2^ compared with that of the included cohort of 27.5 (4.3) kg m^−2^ (*P *< 0.001).

Characteristics of this cohort are shown in Table 1. Although men were taller and heavier than women there was only a small difference in BMI between sexes at age 60–64. Most of the participants fell into the overweight category, 25 ≤ BMI < 30 kg m^−2^ (World Health Organization, 2000). Women had lower hip and spine BMD scores compared with men.

**Table 1:** Characteristics of the MRC NSHD participants with hip and spine mode data at age 60–64 years (*n = *1511)

	Men	Women	*P*‐value
Sex; *n* (%)	729 (48.2)	782 (51.8)	
Age (years) at CRF visit	63.2 (1.17)	63.3 (1.09)	0.11
Height (m)	1.75 (0.06)	1.62 (0.06)	< 0.001
Weight (kg)	85.2 (12.8)	71.5 (12.4)	< 0.001
BMI (kg m^−2^)	27.7 (3.9)	27.2 (4.6)	0.02
Total hip BMD (g cm^−2^)	1.00 (0.14)	0.87 (0.13)	< 0.001
Spine BMD (g cm^−2^)	1.05 (0.19)	0.94 (0.16)	< 0.001

Values shown are mean (SD) apart from the number of participants, *n*. *P*‐value for formal test of sex difference.

### Hip shape

**Figure 2:**
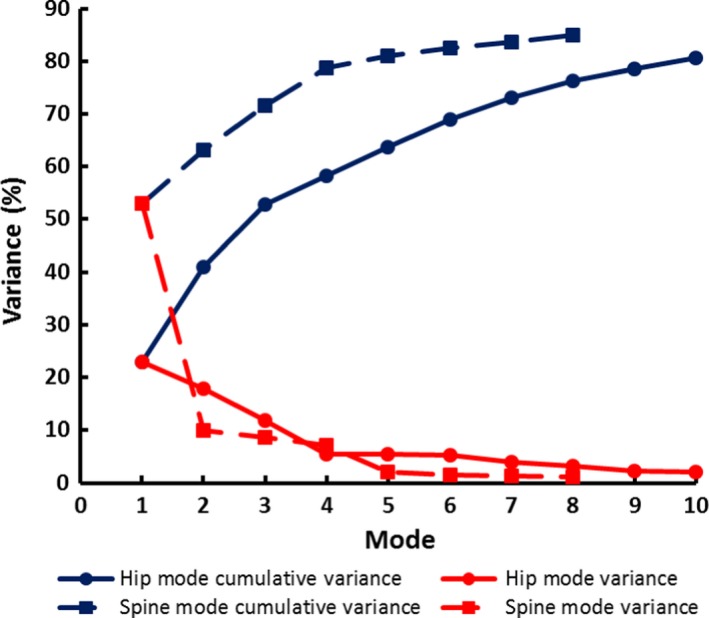
Scree plots of hip and spine PCA data show the total variance and the cumulative variance described by each mode. Changes in the gradient of the curve help to guide how many modes to include in further analyses.

**Figure 3:**
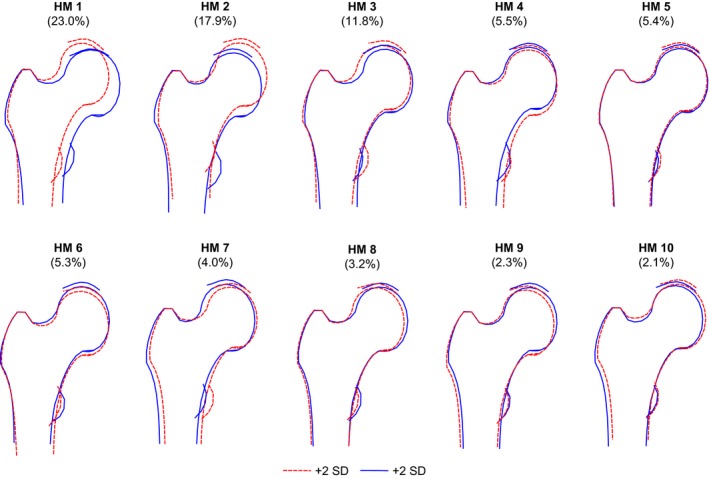
The variation in hip shape detected by hip shape modes 1–10 shown as ± 2 SD from the mean of zero for the whole cohort. Full descriptions of the features identified by each mode may be found in Tables S1–S3 and Figures S1–S4.

A scree plot for hip shape modes (HM) 1–10 is shown in Fig. 2. These first 10 modes represented 80.6% of variation in shape with HM1–HM3 accounting for the majority (52.8% of variation). The largest mode, HM1, contained 23.0% and the smallest chosen for analysis was HM10, which contained 2.1%. Each of the subsequent modes accounted for less than 2% of the variance. Figure 3 shows the variation in hip shapes shown by each of HM1–HM10. More detailed descriptions of these images and the main features identified by each mode may be found in Supporting Information Tables S1–S3and Figures S1–S4. Table 2 shows that there were statistically significant differences in hip shapes between men and women in all modes apart from HM7 and HM5. Women had negative mean scores, whereas men had positive mean scores for HM1, 2, 4, 6 and 8. Conversely, women had positive mean scores and men had negative mean scores for HM 3, 9 and 10. Positive scores for HM1 and HM2 in men represented a wider and shorter femoral neck, smaller neck‐shaft angle and increased presence of osteophytes compared with women. Negative scores for men in HM3 and HM9 described a loss of the femoral head/neck curvature not seen in women. The negative mean scores for women for HM4, 6 and 8 were linked with a smaller neck‐shaft angle, flattening of the femoral head, increased acetabular coverage of the femoral head and superior osteophytosis, possibly indicating an increased prevalence of femoracetabular impingement. Adjustment of the correlations for height resulted in a positive association between female sex and HM5, whereas associations between sex and HM6, 8 and 9 disappeared. Subsequent adjustment for weight had no further effect. Adjustment for BMI did not change the original unadjusted associations. The effects of these adjustments may be found in Table S1–S3 and Figures S1–S4 These comparisons suggest that the sex differences found for HM5, HM6, HM8 and HM9 may be explained by height.

**Table 2:** Sex differences in hip modes

Hip Mode	Men	Women	*P*‐value
	Mean (SD)	Mean (SD)	
HM1	0.22 (1.01)	−0.18 (0.95)	< 0.001
HM2	0.20 (1.01)	−0.19 (0.93)	< 0.001
HM3	−0.27 (1.02)	0.22 (0.92)	< 0.001
HM4	0.24 (1.06)	−0.20 (0.9)	< 0.001
HM5	0.03 (1.04)	−0.02 (0.97)	0.3
HM6	0.19 (0.96)	−0.18 (1.00)	< 0.001
HM7	0.00 (0.98)	0.00 (1.02)	0.99
HM8	0.13 (1.00)	−0.12 (0.99)	< 0.001
HM9	−0.13 (1.01)	0.14 (0.97)	< 0.001
HM10	−0.37 (0.94)	0.34 (0.93)	< 0.001

*P‐*values were obtained from *t*‐tests.

Finding such clear sex differences supported our decision to run all analyses stratified by sex. Partial correlations (adjusting for CRF) showed that three associations were consistent for both men and women with height (positive HM6, negative HM9) or weight (positive HM2). All other linear associations between hip modes and markers of body size differed between women and men, although half of the correlations calculated were very weak in men and more than half in women (Table 3). Greater weight was associated with more positive HM2 scores, which reflected a shorter, wider femoral neck and larger greater and lesser trochanters. HM2 was negatively correlated with height in men, but not in women, and showed the strongest correlation in both sexes with BMI. Greater height was accompanied by a flattening of the curve between superior head and neck and a larger lesser trochanter (increasing HM6) while decreasing values for HM9 indicated a widening and shortening of the femoral neck.

**Table 3:** Partial correlations (adjusted for CRF) between hip modes 1–10 and height, weight, BMI and total hip BMD, by sex

Hip mode	Men				Women			
	Height	Weight	BMI	Total hip BMD	Height	Weight	BMI	Total hip BMD
HM1	−0.02	−0.04	−0.04	−0.01	−0.08	0.01	0.03	−0.06
HM2	−**0.11**	**0.13**	**0.19**	−0.06	−0.02	**0.17**	**0.19**	−0.02
HM3	−0.03	−0.06	−0.04	0.02	−0.04	0.02	0.04	**0.13**
HM4	−0.09	−**0.15**	−**0.12**	−0.07	−0.07	0.01	0.04	0.02
HM5	**0.11**	0.04	0.00	**0.13**	0.09	**0.12**	0.07	0.07
HM6	**0.24**	**0.18**	0.07	0.06	**0.19**	0.07	−0.01	−0.03
HM7	0.02	0.07	0.07	0.03	−0.01	0.06	0.06	−0.01
HM8	0.07	**0.13**	**0.10**	**0.16**	0.05	−0.06	−0.09	0.07
HM9	−**0.13**	−**0.16**	−**0.10**	0.01	−0.09	−0.06	−0.02	−0.03
HM10	−**0.12**	0.03	**0.10**	0.01	−0.05	0.01	0.03	−0.03

Partial correlations with magnitudes 0.1 or greater are highlighted in bold to assist in recognising where the associations primarily lie.

The strength and direction of the linear relationships between hip shape and total hip BMD measurements varied across hip modes (Table 3). Among men, there were weak positive correlations with BMD between HM5 (*r = *0.13) and HM8 (*r = *0.16), together describing a slight outward and downward movement of the femoral head and larger osteophytes, whereas in women HM3 showed the strongest association with BMD (*r = *0.13), suggesting a smaller neck‐shaft angle and greater acetabular coverage with increasing BMD.

### Spine shape

**Figure 4:**
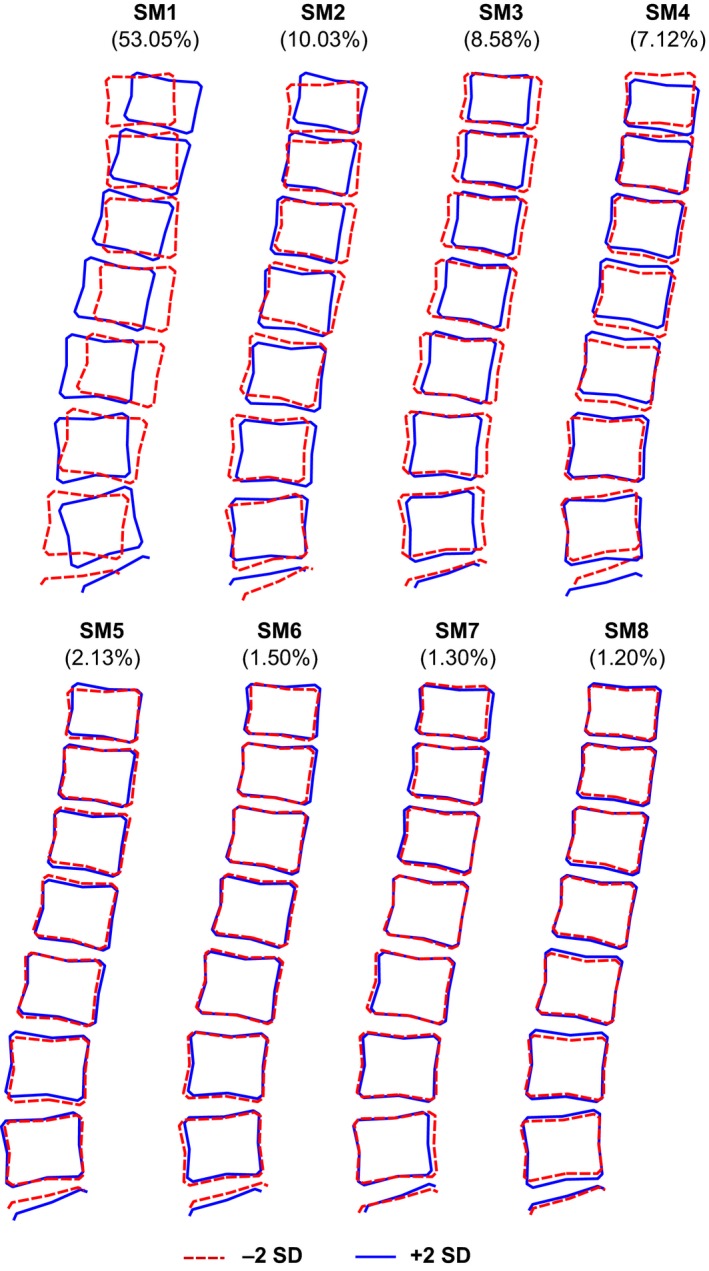
The variation in lumbar spine shape and the percentage variance detected by spine modes 1–8 shown as ± 2 SD from the mean of zero for the whole cohort. Modes 1 and 2 are very similar to previous descriptions we have called curviness and evenness. Full descriptions of the features identified by each mode may be found in Tables S1–S3 and Figures S1–S4.

From the scree plot in Fig. 2 eight spine shape modes (SM) were chosen for further analysis. Together, these eight SM describe 84.9% of the variance in the dataset; the largest mode SM1 accounts for 53.0% and the smallest, SM8, 1.2%. Figure 4 shows the variation in spine shapes represented by each of the modes SM1–SM8. Descriptions of the main features identified by these modes may be found in Tables S1–S3 and Figures S1–S4.

Similarly to the hip, significant differences in spine mode scores between men and women justified our use of sex‐stratified analyses. Significant differences were seen between men and women described by SM1, SM3, SM6 and SM8 (Table 4). Women had a positive mean score for SM1, SM3 and SM8, whereas men had negative mean scores for these modes, and women had a negative mean score for SM6 whereas men had a positive mean score. The biggest difference was seen in SM3; the mean shapes of men and women fell approximately a whole standard deviation apart. This mode described anterior‐posterior vertebral body diameters (a‐p diameter) relative to vertebral body heights; these latter were all similar because overall size was removed by scaling. Men, therefore, with negative scores for SM3 had larger a‐p diameters relative to vertebral height compared with women. Adjusting for height only slightly attenuated the association with SM3 but removed the association with sex in SM1 and resulted in a negative association with female sex in SM4. Subsequent adjustment for body weight resulted in a further small reduction in the association with SM3, but adjustment for BMI alone had no effect. The effects of these adjustments may be found in Tables S1–S3 and Figures S1–S4. On the whole, women had a slightly more lordotic spine (positive SM1) with relatively smaller but caudally increasing a‐p diameters (positive SM3, negative SM6). Men, on the other hand, were more likely to have a straighter spine (negative SM1) with relatively larger and more uniform a‐p diameters (negative SM3, positive SM6). Additionally, men had marginally smaller relative vertebral heights at the second to fourth lumbar levels (L2–L4) but larger disc spaces compared with women (negative SM8).

**Table 4:** Sex differences in spine shape modes

Spine mode	Men	Women	*P*‐value
	Mean (SD)	Mean (SD)	
SM1	−**0.08 (0.97)**	**0.07 (1.03)**	**0.003**
SM2	0.02 (1.01)	−0.02 (0.98)	0.53
SM3	−**0.50 (0.98)**	**0.47 (0.77)**	**< 0.001**
SM4	0.05 (0.97)	−0.04 (1.02)	0.11
SM5	0.04 (1.00)	−0.03 (1.00)	0.17
SM6	**0.19 (0.97)**	−**0.18 (0.99)**	**< 0.001**
SM7	0.03 (1.04)	−0.03 (0.96)	0.26
SM8	−**0.26 (1.00)**	**0.24 (0.93)**	**< 0.001**

Significant sex differences are highlighted in bold and *P‐*values arise from *t*‐tests.

Again using *r *> 0.1 as an arbitrary threshold to aid interpretation, there were some patterns of linear correlation between spine modes and markers of body size that were consistent in both men and women in SM3, 6 and 8. There were weak negative correlations between SM3 and SM6 and body weight and BMI (but not height) (Table 5), indicating that heavier individuals have larger a‐p diameters (negative SM3), which increased caudally and presented with a smaller L4/L5 disc space (negative SM6). In both sexes there was a weak negative correlation between SM8 and height, indicating relatively smaller vertebral body heights with larger intervertebral disc spaces in taller individuals. However, in women, a higher BMI was associated with larger vertebral body heights and smaller disc spaces (positive SM8). In women there was a weak positive correlation between height and SM2, indicating that taller women appeared to have more uniform spinal curvatures. In both sexes, SM3 was negatively correlated with BMD, although slightly more strongly in men; hence greater BMD was associated with larger a‐p diameters (negative SM3). Men also had a negative correlation with SM4 and therefore greater BMD was associated with ‘snakier’ curvatures with smaller L4/L5 disc space.

**Table 5:** Partial correlations (adjusted for CRF) between spine modes 1–8 and height, weight, BMI and lumbar spine BMD, by sex

Spine mode	Men				Women			
	Height	Weight	BMI	Spine BMD	Height	Weight	BMI	Spine BMD
SM1	−0.04	−0.02	0.00	0.03	0.01	0.00	−0.01	0.06
SM2	0.04	−0.05	−0.08	0.03	**0.11**	0.01	−0.04	0.08
SM3	−0.09	−**0.17**	−**0.13**	−**0.23**	−0.04	−**0.13**	−**0.11**	−**0.10**
SM4	−0.04	−0.02	0.00	−**0.17**	−0.04	0.05	0.07	−0.08
SM5	0.02	−0.02	−0.03	−0.02	−0.06	−0.09	−0.07	−0.09
SM6	−0.07	−**0.15**	−**0.12**	−0.06	0.01	−**0.10**	−**0.10**	−0.05
SM7	−0.06	−0.07	−0.04	0.06	0.05	0.03	0.01	0.02
SM8	−**0.10**	0.01	0.06	0.08	−**0.13**	0.07	**0.13**	0.07

Associations with magnitudes 0.1 or greater have been highlighted in bold for clarity.

### Relationships between hip and spine shapes

Linear relationships between hip and spine shapes were weak and inconsistent in both sexes with no correlation coefficients of greater magnitude than 0.14 for men and none over 0.08 for women (Table 6). There was a weak positive correlation between HM3 and SM1 in men (*r = *0.10), suggesting that men with flatter or more kyphotic spinal curvatures (negative SM1) were more likely to have hips with relatively larger femoral heads, osteophytes, and a wider and flatter femoral neck (negative HM3). In men there was also a weak negative correlation between HM2 and SM2 (*r = *−0.13), suggesting that men with more uniform spinal curvatures (positive SM2) had a relatively narrower and flatter femoral neck with greater internal rotation in their hip joint (negative HM2). Other correlations in men were between higher modes describing more subtle shape variations.

**Table 6:** Partial correlations (adjusted for CRF) between hip modes (HM1–10) and spine modes (SM1–8) in (a) men and (b) women

(a)								
Modes	SM1	SM2	SM3	SM4	SM5	SM6	SM7	SM8
HM1	0.01	−0.04	0.01	−0.03	0.04	0.03	−0.03	0.01
HM2	0.01	−**0.13**	−0.07	0.02	−0.01	−0.02	−0.03	0.03
HM3	**0.10**	−0.07	0.05	−0.06	−0.03	0.00	0.01	−0.06
HM4	0.03	0.00	−0.01	0.01	−**0.11**	0.01	0.03	**0.14**
HM5	−0.06	−0.05	0.01	0.07	−0.01	−0.02	0.03	−0.02
HM6	−0.01	−0.02	−0.09	−0.05	0.06	−0.09	−0.05	−0.03
HM7	−0.06	0.01	0.08	0.04	0.04	−0.04	−0.04	−0.03
HM8	−0.03	−0.03	0.08	−0.03	−0.06	−0.04	0.04	0.03
HM9	0.03	0.08	0.07	0.01	0.02	0.02	0.09	0.04
HM10	−0.07	−0.03	−0.02	0.01	0.08	−0.02	0.00	**0.12**

(b)								
Modes	SM1	SM2	SM3	SM4	SM5	SM6	SM7	SM8
HM1	−0.07	−0.07	−0.01	−0.03	0.01	0.04	−0.05	0.01
HM2	−0.02	−0.01	−0.05	0.06	−0.03	0.01	−0.03	0.08
HM3	−0.07	−0.06	0.05	−0.03	−0.03	0.04	−0.02	0.02
HM4	0.01	0.04	−0.02	0.03	−0.03	−0.07	−0.03	0.06
HM5	0.00	0.01	0.02	0.03	0.05	0.03	−0.04	0.00
HM6	−0.01	0.01	−0.07	−0.05	0.02	−0.04	−0.04	0.00
HM7	−0.07	0.00	−0.02	−0.02	−0.02	−0.04	0.02	−0.04
HM8	−0.04	−0.02	0.08	−0.03	0.08	0.02	0.01	−0.01
HM9	−0.03	−0.04	0.04	−0.03	0.08	0.01	−0.02	0.06
HM10	−0.01	0.03	0.04	−0.01	0.00	−0.03	0.01	0.03

Partial correlations with magnitudes 0.1 or greater have been highlighted to aid analysis.

## Discussion

This study is the first to examine shape models from different regions in the same people. It is also the largest study of its kind to date and is unique in that, although it is cross‐sectional, because it is a birth cohort all the individuals are the same age, within a small interval to allow time for imaging. Although the narrow age range of participants prevents us from investigating how relationships differ by age, it does allow us to explore relationships free from the strong confounding effect of age (which is a major, often overlooked, limitation of studies with age‐heterogeneous samples). At this stage we took no account of morbidities, pain or pathology and this, therefore, represents the shapes of the hips and spines in a reasonable cross‐section of the community aged in their early 60s as represented by the cohort (Stafford et al. 2013). These baseline data will enable comparisons to be made with different age groups in future studies, and with morbidities and lifestyle factors, to try to identify risk factors for hip and spine disorders and modifiable factors to reduce risk.

The results demonstrate clear differences in the shapes of both hips and spines between men and women in their early 60s. These differences could be attributable to sex differences in both developmental and degenerative processes but at this stage it is not possible to separate these. We had hypothesised that the shapes of the hip and spine would be strongly associated, but this was not the case when using SSM to characterise these shapes, as shown by the weak correlations between hip and spine mode scores in both men and women. Correlations between higher modes describe subtle variations in shape whose clinical or anatomical relevance is unknown. Correlations were found for both hip and spine shapes with height, weight, BMI and local BMD but, again, these associations were not strong and differed between men and women. Taller women had more uniform spinal curvatures, larger intervertebral disc spaces, a wider and shorter femoral neck with a flattening of the curve between the superior femoral neck and head and a larger lesser trochanter. In contrast, the only difference in spine shapes between tall and short men was a larger disc space in taller men. Increasing weight was also associated with a wider and shorter femoral neck in men and women and with increasing spinal a‐p diameters, probably implying larger vertebral cross‐sections, as a‐p diameter was also positively correlated with BMD.

A difference in lumbar lordosis between men and women has been in question for some time and the results of previous studies are conflicting (Youdas et al. 2006; Consmüller et al. 2012; Endo et al. 2012). Vialle et al. (2005) and colleagues examined radiographs of 300 individuals aged 35 (± 12) years old and found females to have on average a 5° greater lordosis than men. They found no association with age when controlling for sex. Using SSM in a sample of 30 individuals we previously found no sex differences in overall curvature (Pavlova et al. 2014), although these individuals were younger than in the current study. In this larger sample, however, we have found that, on average, women had a slightly more lordotic curve than men. This could be due simply to having larger numbers, giving us more statistical power to detect a smaller effect, or it could be due to the inclusion of the lower three thoracic vertebrae into the model, providing more curvature. Controlling for height, however, explained this sex difference. Some of the lack of association might be due to the scans being taken supine rather than in a weight‐bearing posture. While the shape of the hips may be less affected between standing and lying, the shape of the spine changes measurably on going from standing to supine. This conclusion is supported by a recent study of 200 individuals which found a significantly larger lordosis angle in women than men while standing but not while lying supine (Bailey et al. 2016). Although in previous studies we showed that an element of an intrinsic shape can still be identified between these postures (Meakin et al. 2009), more associations between joint shapes may be evident in the natural weight‐bearing position. To do this, subjects would need to be scanned in an upright scanner where both hips and spine could be imaged at the same time.

Previous SSMs of the lumbar region have consistently identified two primary modes of variation we have called curviness and evenness (Meakin et al. 2008, 2009; Pavlova et al. 2014). Curviness describes the overall curvature from lordotic to almost straight, while evenness describes whether curvature appears uniformly along the lumbar spine or is found predominantly in the lower lumbar region. Because the current study contains vertebrae up to T10 but not the sacrum, the modes identified differ slightly from previous findings. The model has to describe vertebral rotation and flexion‐extension at T10–L1 and hence does not identify as clearly the features previously found in models containing L1–S1. The largest differences here were in vertebral size, and these are not explained by height or weight differences between men and women. Similarly, a study using dissected thoracic and lumbar vertebrae from 240 individuals and a comparable measurement of a‐p diameter, termed vertebral body length, reported consistently larger average values in males compared with females, although they did not formally test for sex differences (Masharawi et al. 2008). Other differences reaching statistical significance were more subtle variations in disc and vertebral dimensions, described by the higher modes.

Statistical shape modelling provides a unique way of quantifying shape variations and of exploring variations that happen in a coordinated way. Unlike geometrical measures (e.g. femoral neck length and width), which are often highly correlated, the principal components analysis results in modes that are linearly unrelated and normally, or very close to normally, distributed. Although these modes of variation may be harder to interpret than geometrical measures, they do convey several advantages for identifying shape variations that are commonly found together. Although DXA images do not have such high resolution as plain radiographs, we have shown in previous studies that similar precision in point placement can be achieved using the two imaging modalities (Gregory et al. 2005). For imaging the hip, the feet are internally rotated and strapped to a support, which makes positioning very reproducible. The spine was imaged in the same posture without moving the individual in five of the six CRFs, as these had a scanner with a C‐arm that could be rotated to record a lateral image. The scanner in one of the CRFs had a fixed arm, however, and we used partial correlations to adjust for CRF, to examine the relationship between hip and spine shape mode scores and correlations of mode scores with height, weight BMD and BMI. Unadjusted correlation coefficients were very similar to the partial correlation coefficients (see Tables S1–S3 and Figures S1–S4).

Despite all the above precautions, rotation may still affect the measurement of BMD in both hip and spine (Cheng et al. 2001) and also may result in apparent foreshortening of the femoral neck, which in turn may affect the calculated mode scores. DXA imaging is, in general, much more reliable than plain radiography, unless used with a positioning device, due to the normal care taken with leg positioning to optimise BMD measurements. The feet are held in internal rotation and, consequently, any rotation in the femoral neck will represent normal variation that is found in the general population. We carried out an earlier pilot study in which a set of femora were each rotated about the long axes; little variation in shape measures was found, provided rotation did not exceed a few degrees. This might be expected from a sine variation in perspective in which even a 5° rotation results in shortening by less than 3%. Similarly, although rotational conditions such as scoliosis may affect the measured lordosis, studies suggest the effect is small. Legaye et al. (1998) found little or no difference in the lumbar lordosis (from L5–T12) of scoliotic patients (*n = *66) and controls (*n = *49). Furthermore, in a recent review of studies of lumbar lordosis (Been & Kalichman, 2014), scoliosis was not identified as one of the spinal conditions to have a clear association with lumbar lordosis, whereas spondylolysis and isthmic spondylolisthesis were associated. The NHANES study of 6594 adults in the USA reported a scoliosis prevalence of 8.3% in their population of 25‐ to 74‐year‐olds. Although prevalence increased with age among women, it did not change significantly in men (Carter & Haynes, 1987).

In conclusion, in men and women entering their seventh decade there are small but statistically significant differences in the shapes of their hips and spines. Associations with height, weight, BMI and BMD are small and correspond to subtle variations, the anatomical significance of which is not yet clear. Correlations between hip and spine shapes are small.

## Funding

The NSHD is funded by the UK Medical Research Council.

S.G.M., R.C., R.J.H. and D.K. are supported by the UK Medical Research Council (Programme codes: MC_UU_12019/1, MC_UU_12019/2 and MC_UU_12019/4). This project was funded by the UK Medical Research Council (Grant MR/L010399/1), which supported S.G.M., A.V.P. and F.R.S.

## Author contributions

A.V.P., F.R.S., S.G.M., J.S.G., R.J.B., K.R.M., R.J.H. and R.C. contributed to the design of the study and the interpretation of the data, critically revised the manuscript and approved the final article. .

J.E.A. directed the imaging arm of the NSHD and is curator of the images. She critically revised the manuscript and approved the final article.

D.K. is director of the NSHD and contributed to the design of the study and the interpretation of the data, critically revised the manuscript and approved the final article.

R.M.A. contributed to the design of the study, the interpretation of the data, drafted the first version of the manuscript, performed the critical revisions from the comments returned by co‐authors and approved the final article.

## Supporting information


**Table S1.** Unadjusted **c**orrelations between hip modes 1–10 and height, weight, BMI and total hip BMD, by sex.
**Table S2.** Unadjusted correlations between spine modes 1–8 and height, weight, BMI and lumbar spine BMD, by sex.
**Table S3.** Unadjusted correlations between hip modes (HM1–10) and spine modes (SM1–8) in (a) men and (b) women.
**Fig. S1.** Effects of adjustment for height, weight and BMI on associations between hip mode scores and sex.
**Fig. S2.** Effects of adjustment for height, weight and BMI on associations between spine mode scores and sex.
**Fig. S3.** A description of the features varying in a coordinated fashion as identified by the hip mode scores HM1–HM10. The average score for each mode of the whole cohort is zero and positive and negative scores are described relative to the average.
**Fig. S4.** A description of the features varying in a coordinated fashion as identified by the spine mode scores SM1–SM8. The average score for each mode of the whole cohort is zero and positive and negative scores are described relative to the average.
