# Supplementary material for: Statistical shape modelling of hip and lumbar spine morphology and their relationship in the MRC National Survey of Health and Development
Source: J Anat. 2017 May 31;231(2):248–59. doi: 10.1111/joa.12631 (PMC5522893; doi:10.1111/joa.12631)
Supplement: Supplementary file 1 — Table S1. Unadjusted correlations between hip modes 1–10 and height, weight, BMI and total hip BMD, by sex. Table S2. Unadjusted correlations between spine modes 1–8 and height, weight, BMI and lumbar spine BMD, by sex. Table S3. Unadjusted correlations between hip modes (HM1–10) and spine modes (SM1–8) in (a) men and (b) women. Fig. S1. Effects of adjustment for height, weight and BMI on associations between hip mode scores and sex. Fig. S2. Effects of adjustment for height, weight and BMI on associations between spine mode scores and sex. Fig. S3. A description of the features varying in a coordinated fashion as identified by the hip mode scores HM1–HM10. The average score for each mode of the whole cohort is zero and positive and negative scores are described relative to the average. Fig. S4. A description of the features varying in a coordinated fashion as identified by the spine mode scores SM1–SM8. The average score for each mode of the whole cohort is zero and positive and negative scores are described relative to the average. [file JOA-231-248-s001.pdf]

Statistical shape modelling of hip and lumbar spine morphology and their relationship in the MRC National Survey of Health and Development.

Supporting Information

Anastasia V. Pavlova, Fiona R. Saunders, Stella G. Muthuri, Jennifer S. Gregory, Rebecca J. Barr, Kathryn R. Martin, Rebecca J. Hardy, Rachel Cooper, Judith E. Adams, Diana Kuh, and Richard M. Aspden

Contents

Table S1. Unadjusted **c**orrelations between hip modes 1-10 and height, weight, BMI and total hip BMD, by sex

Table S2. Unadjusted correlations between spine modes 1-8 and height, weight, BMI and lumbar spine BMD, by sex.

Table S3. Unadjusted correlations between hip modes (HM1-10) and spine modes (SM1-8) in (a) men and (b) women.

Figure S1. Effects of adjustment for height, weight and BMI on associations between hip mode scores and sex

Figure S2. Effects of adjustment for height, weight and BMI on associations between spine mode scores and sex

Figure S3. A description of the features varying in a coordinated fashion as identified by the hip mode scores HM1-HM10. The average score for each mode of the whole cohort is zero and positive and negative scores are described relative to the average.

Figure S4. A description of the features varying in a coordinated fashion as identified by the spine mode scores SM1-SM8. The average score for each mode of the whole cohort is zero and positive and negative scores are described relative to the average.

Table S1. Unadjusted correlations between hip modes 1-10 and height, weight, BMI and total hip BMD, by sex. Correlations with magnitudes greater than 0.1 have been emboldened to assist in recognising where the associations primarily lie.

Hip mode	Men				Women			
	Height	Weight	BMI	Total hip BMD	Height	Weight	BMI	Total hip BMD
HM1	-0.02	-0.04	-0.04	-0.01	-0.08	0.01	0.04	-0.06
HM2	**-0.11**	**0.13**	**0.19**	-0.07	-0.01	**0.18**	**0.19**	-0.01
HM3	-0.04	-0.06	-0.04	0.00	-0.05	0.02	0.04	**0.12**
HM4	-0.09	**-0.15**	**-0.12**	-0.06	-0.07	0.01	0.04	0.02
HM5	**0.11**	0.05	0.00	**0.14**	0.09	**0.12**	0.07	0.07
HM6	**0.24**	**0.18**	0.07	0.06	**0.18**	0.07	-0.01	-0.04
HM7	0.02	0.07	0.07	0.03	0.00	0.06	0.06	-0.01
HM8	0.07	**0.13**	**0.10**	**0.17**	0.05	-0.07	-0.09	0.07
HM9	**-0.13**	**-0.16**	**-0.10**	0.00	-0.09	-0.07	-0.03	-0.03
HM10	**-0.12**	0.03	**0.10**	0.01	-0.05	0.01	0.03	-0.02

Table S2. Unadjusted correlations between spine modes 1-8 and height, weight, BMI and lumbar spine BMD, by sex. Associations greater than 0.1 have been highlighted in bold for clarity.

	Men				Women			
Spine mode	Height	Weight	BMI	Spine BMD	Height	Weight	BMI	Spine BMD
SM1	-0.04	-0.02	0.00	0.02	0.00	-0.01	-0.01	0.05
SM2	0.05	-0.05	-0.08	0.03	**0.11**	0.02	-0.03	0.07
SM3	-0.09	**-0.16**	**-0.13**	**-0.23**	-0.03	**-0.13**	**-0.12**	-0.09
SM4	-0.04	-0.02	0.00	**-0.17**	-0.05	0.05	0.07	-0.08
SM5	0.01	-0.02	-0.03	-0.03	-0.07	-0.09	-0.07	**-0.10**
SM6	-0.07	**-0.15**	**-0.12**	-0.07	0.01	-0.09	-0.09	-0.05
SM7	-0.06	-0.07	-0.05	0.07	0.05	0.03	0.02	0.02
SM8	-0.09	0.01	0.06	0.06	**-0.13**	0.07	**0.13**	0.06

Table S3. Unadjusted correlations between hip modes (HM1-10) and spine modes (SM1-8) in (a) men and (b) women. Values greater than 0.1 have been highlighted to aid analysis

a)

Modes	SM1	SM2	SM3	SM4	SM5	SM6	SM7	SM8
HM1	0.02	-0.05	0.01	-0.03	0.05	0.03	-0.03	0.01
HM2	0.02	**-0.13**	-0.07	0.02	0.00	-0.02	-0.02	0.03
HM3	**0.11**	-0.07	0.05	-0.06	-0.03	0.00	0.01	-0.06
HM4	0.02	0.00	0.00	0.01	**-0.12**	0.00	0.03	**0.14**
HM5	-0.07	-0.04	0.01	0.07	-0.02	-0.03	0.02	-0.02
HM6	-0.01	-0.02	-0.09	-0.05	0.06	-0.09	-0.05	-0.03
HM7	-0.06	0.00	0.07	0.05	0.04	-0.05	-0.04	-0.03
HM8	-0.04	-0.03	0.07	-0.03	-0.07	-0.05	0.04	0.01
HM9	0.04	0.08	0.07	0.01	0.02	0.02	0.09	0.04
HM10	-0.06	-0.03	-0.02	0.01	0.08	-0.02	-0.01	**0.11**

b)

Modes	SM1	SM2	SM3	SM4	SM5	SM6	SM7	SM8
HM1	-0.07	-0.07	-0.01	-0.03	0.01	0.04	-0.05	0.01
HM2	-0.02	-0.01	-0.05	0.06	-0.03	0.01	-0.03	0.08
HM3	-0.07	-0.05	0.05	-0.02	-0.02	0.03	-0.01	0.03
HM4	0.01	0.03	-0.02	0.03	-0.03	-0.07	-0.04	0.05
HM5	0.00	0.01	0.02	0.03	0.05	0.03	-0.04	0.00
HM6	0.00	0.01	-0.07	-0.05	0.03	-0.04	-0.04	0.01
HM7	-0.07	0.00	-0.02	-0.02	-0.01	-0.04	0.02	-0.04
HM8	-0.03	-0.02	0.09	-0.03	0.07	0.03	0.00	-0.02
HM9	-0.03	-0.04	0.05	-0.03	0.08	0.01	-0.02	0.06
HM10	-0.01	0.03	0.04	-0.02	0.00	-0.03	0.01	0.03

a) Hip modes 1-5

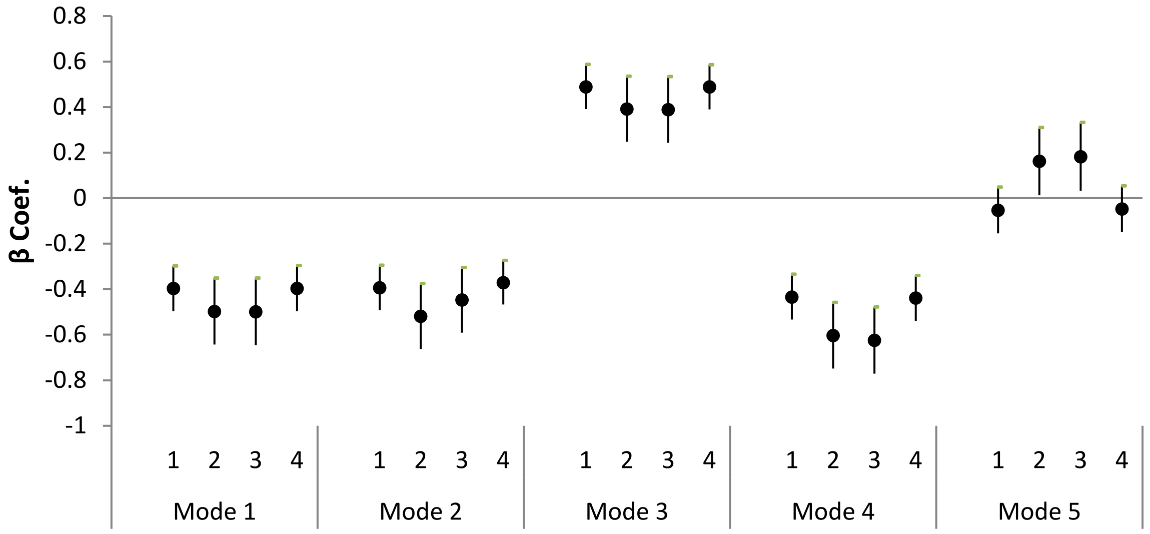

b) Hip modes 6-10

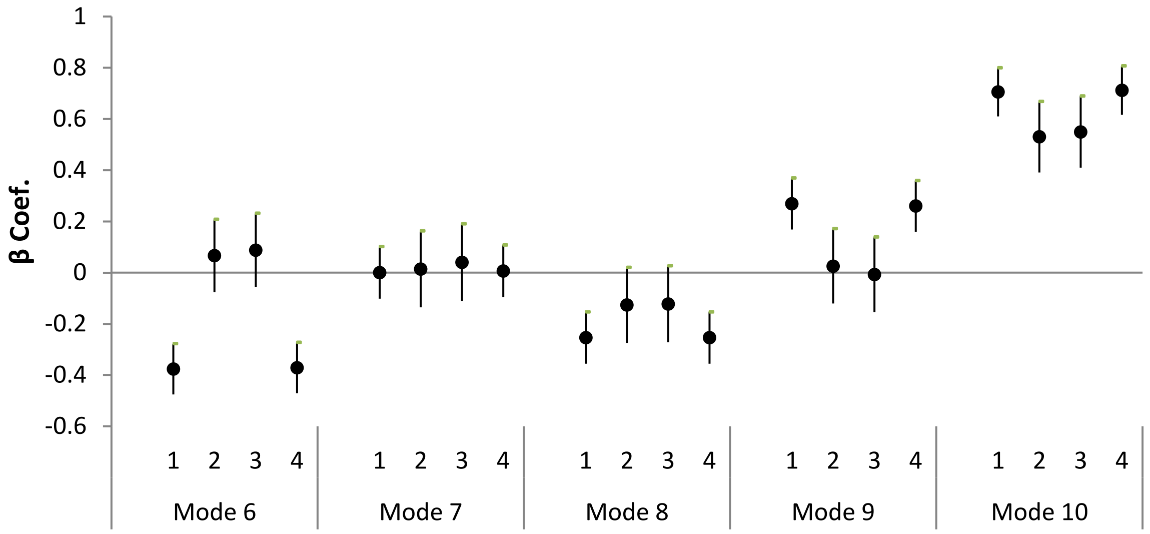

Models: 1, unadjusted; 2, adjusted for height; 3, adjusted for height and weight; 4, adjusted for BMI

Figure S1. Effects of adjustment for height, weight and BMI on associations between hip mode scores and sex. Findings from unadjusted models (Model 1) show associations between sex and hip modes. Except for modes 5 and 7, women had positive scores for modes 3, 9, and 10 but negative scores for modes 1, 2, 4, 6 and 8, as compared with men (Table 2). Adjustment for height (model 2) had the greatest effect on the findings and the association between sex and mode 5 become stronger whereas no associations were then observed between sex and modes 6, 8 and 9. There was little effect of adjusting for BMI (Model 4) whereby similar size estimates to those for model 1 were observed; suggesting that sex-differences found for modes 5, 6, 8 and 9 may be explained by height.

a) Spine modes 1 - 4

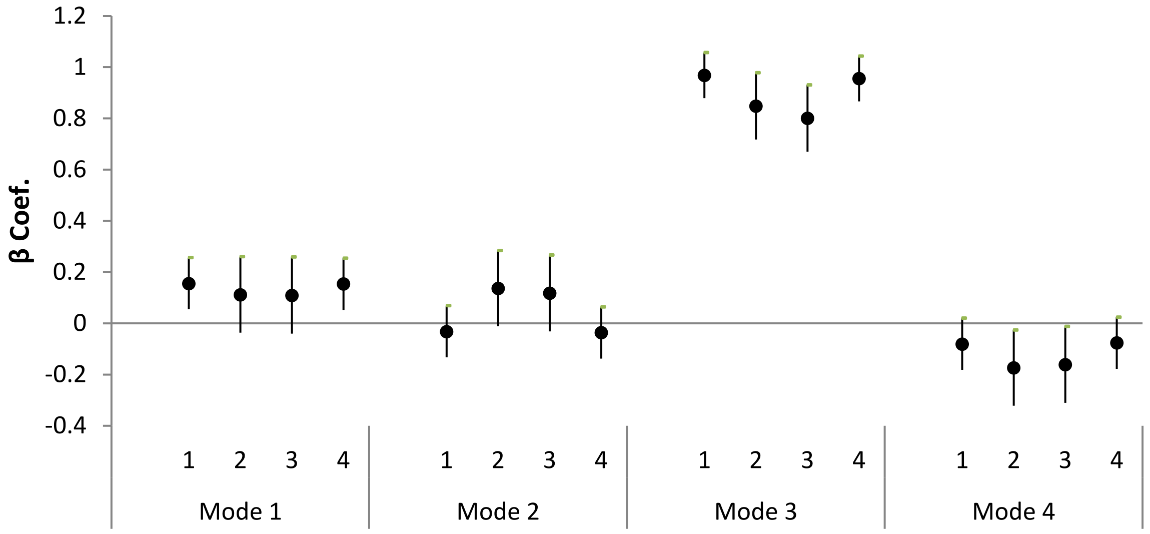

b) Spine modes 5 – 8

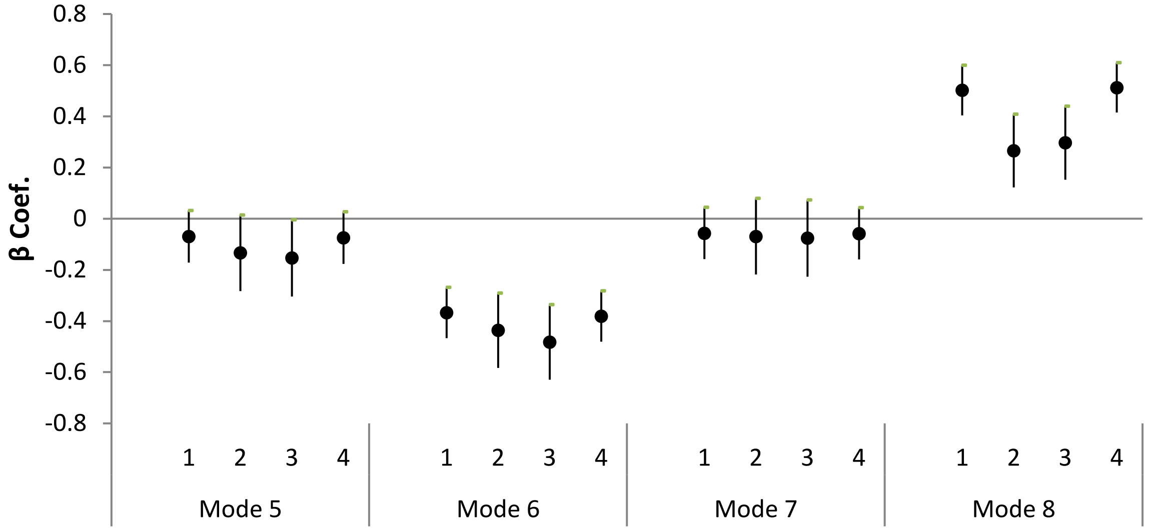

Models: 1, unadjusted; 2, adjusted for height; 3, adjusted for height and weight; 4, adjusted for BMI

Figure S2. Effects of adjustment for height, weight and BMI on associations between spine mode scores and sex. Findings from unadjusted analyses show associations between sex and spine modes 1, 3, 6 and 8. Compared with men, women were more likely to have positive scores for spine modes 1, 3 and 8 but negative scores for mode 6 (Model 1). Adjustment for height slightly attenuated size estimates for mode 1 and the association become null. Conversely, associations between sex and mode 4 become stronger after adjustment for height.

Mode (% of variation)	Description	±2 SD
**1 (23.0%)**	Negative scores<br>• More compact femoral head<br>• Larger neck-shaft angle<br><br>Positive scores<br>• Femoral head migration<br>• increased osteophytes<br>• wider femoral neck<br>• Smaller neck-shaft angle<br><br>• Flattening of the femoral neck from negative to positive scores	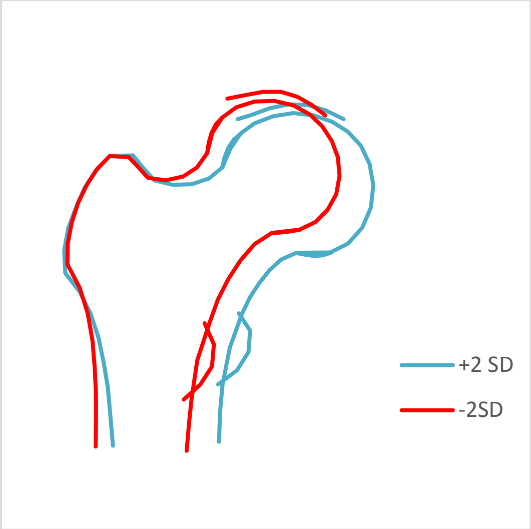
**2 (18.0%)**	Negative scores<br>• Longer femoral neck<br>• Increased external rotation, as shown by lesser trochanter inside the femoral shaft<br>• Loss of femoral head curvature<br><br>Positive scores<br>• Wider greater trochanter<br>• Larger lesser trochanter<br>• Wider femoral head and neck<br>• Superior and Inferior osteophytes	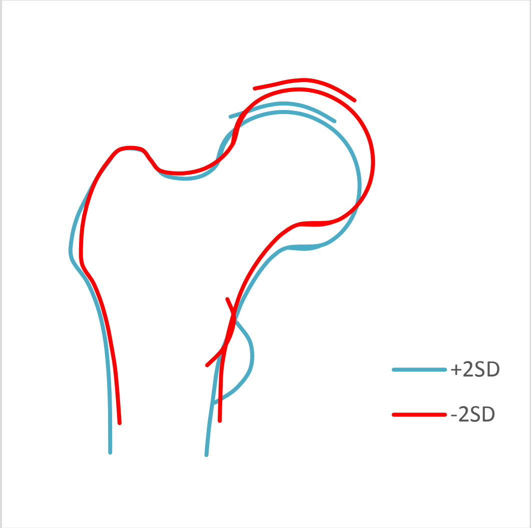

**3 (11.9%)**	Negative scores<br>• Possible external rotation<br>• Bigger femoral head<br>• Loss of femoral head to neck curvature<br>• Increased osteophytes superiorly and inferiorly<br>• Wider femoral neck<br><br>Positive scores<br>• Smaller neck-shaft angle<br>• Greater acetabular coverage	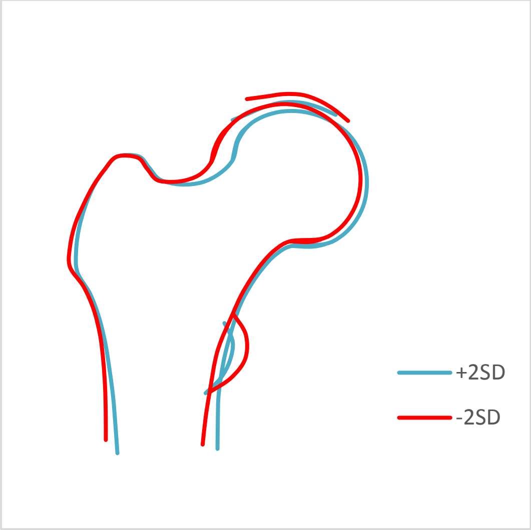
**4 (5.5 %)**	Negative scores<br>• Bigger, flatter femoral head<br>• Wider femoral neck<br>• Smaller neck-shaft angle<br><br>Positive scores<br>• Possible external rotation<br>• Increased inferior osteophytes<br>• Small increase in acetabular coverage	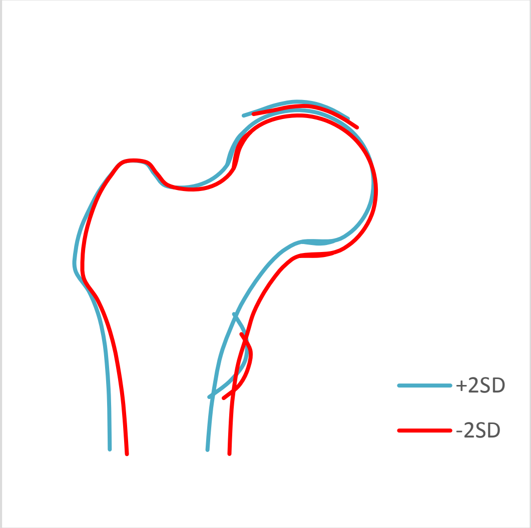

**5 (5.4%)**	Negative scores<br>• Possible external rotation (more of the lesser trochanter visible)<br><br>Positive scores<br>• Slight flattening of the inferior femoral head<br>• Increased osteophytes	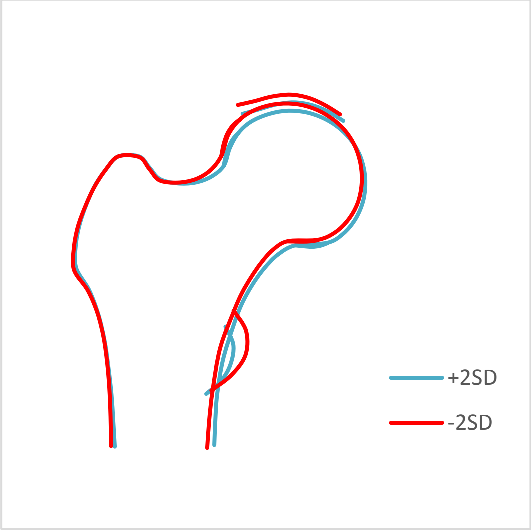
**6 (5.3%)**	Negative scores<br>• Flattening of the femoral head<br>• Change in curve between femoral head and neck<br><br>• Some evidence of external rotation from positive to negative scores	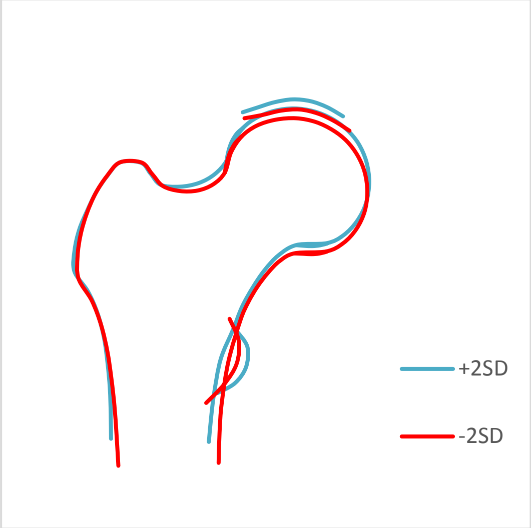

**7 (4.1%)**	Negative scores<br>• Longer femoral neck<br>• More compact femoral head<br>• Increase in osteophytes<br><br>Positive scores<br>• Wider, flatter femoral head<br>• Shorter femoral neck<br>• Slight external rotation	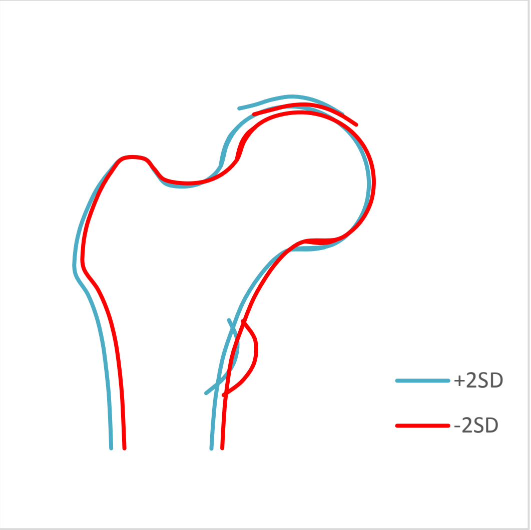
**8 (3.2%)**	Negative scores<br>• Wider, flatter femoral head<br>• Greater acetabular coverage<br>• Larger superior osteophyte<br><br>Positive scores<br>• Slight medial migration of femoral head<br>• Slightly larger lesser trochanter	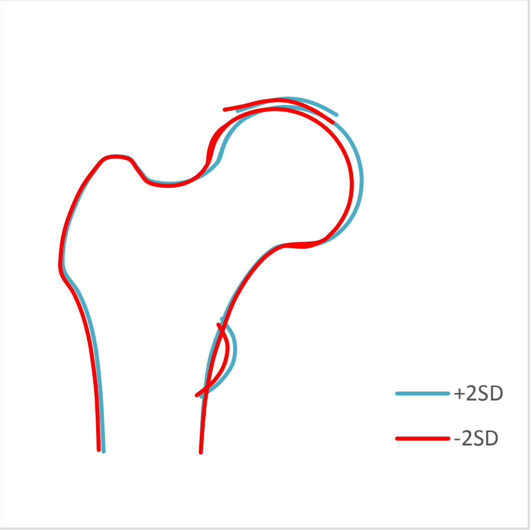

**9 (2.3%)**	Negative scores<br>• Wider femoral neck<br>• Increasing osteophytes<br>• More compact femoral head<br><br>Positive scores<br>• Slight proximo-medial migration of femoral head	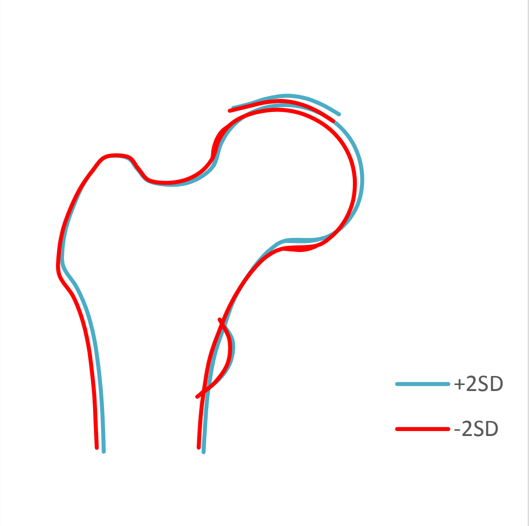
**10 (2.0%)**	Negative scores<br>• Flatter femoral neck curvature<br>• Medial enlargement of femoral head<br>• Narrower femoral shaft<br><br>Positive scores<br>• Wider greater trochanter<br>• Greater acetabular coverage<br>• Narrower neck	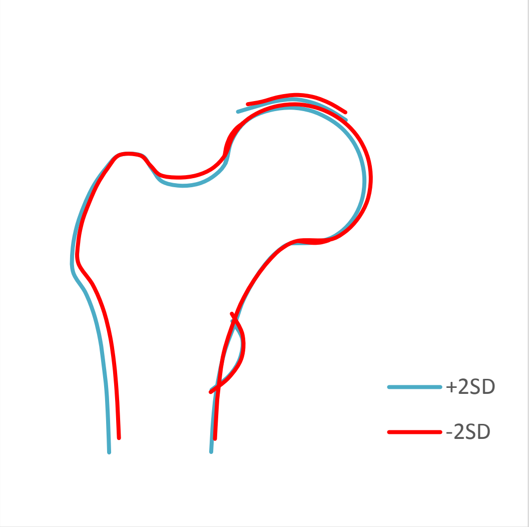

Figure S3. A description of features of the hip joint that vary in a coordinated fashion as identified by the hip mode scores HM1-HM10. The average score for each mode of the whole cohort is zero and positive and negative scores are described relative to the average. The percentage variation is the variance described by each principal component.

Mode (%Var)	Description	±2 SD overlay	-2 SD	+2 SD
**1**<br>**(53.0%)**	**Curviness**<br>Total amount and direction of curvature within the spine from L5 to T10.<br><br>Negative scores:<br>• Flatter lumbar lordosis and a slight kyphosis in thoracic region (T12-T10).<br><br>Positive scores:<br>• Overall greater curvature throughout, increasing lordosis in both lumbar and thoracic sections.	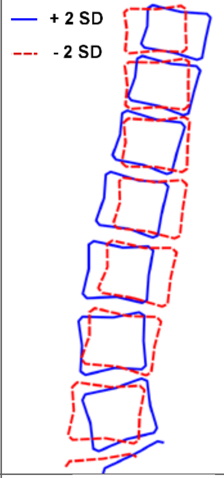	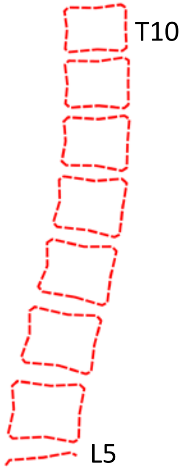	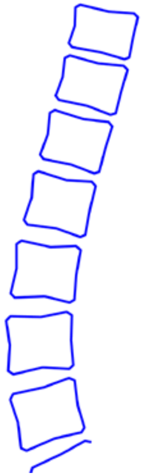
**2**<br>**(10.0%)**	**Evenness**<br>Differences in the distribution of curvature along the length of the spine, with consequent small variations in disc space.<br><br>Negative scores:<br>• Snaking curve with a lumbar lordosis and thoracic kyphosis centred around L1/T12.<br><br>Positive scores:<br>• Increasingly evenly distributed curvature throughout all sections from L5-T10 (superimposed on lordotic, straight or kyphotic overall shape described by SM1).	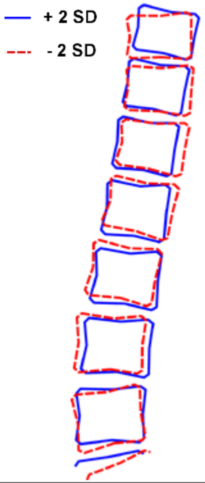	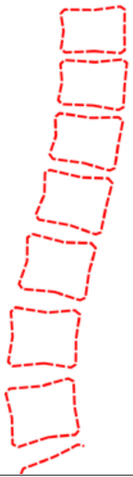	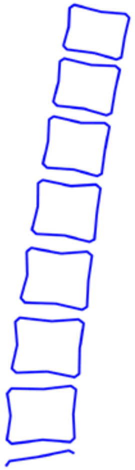

**3 (8.6%)**	Relative anterior-posterior diameter<br><br>Negative scores:<br>• Greater relative vertebral body a-p diameter<br><br>Positive scores:<br>• Smaller relative vertebral body a-p diameter.	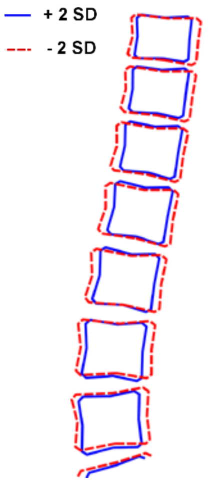	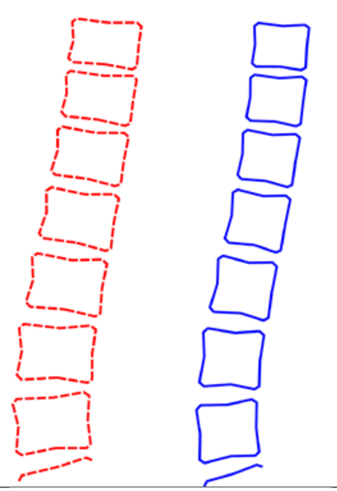
**4 (7.1%)**	A combination of vertebral rotation at L5-L4 and T10 together with changes in disc space<br><br>Negative scores:<br>• Minor snaking of the curvature with greater anti-clockwise rotation at L5, L4, and T10 with smaller caudal disc spaces.<br><br>Positive scores:<br>• More uniform shape with smaller cranial disc spaces.	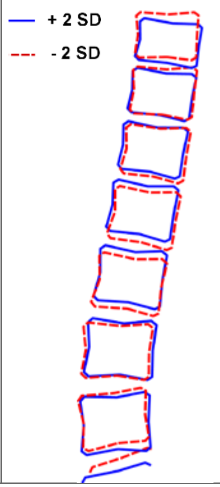	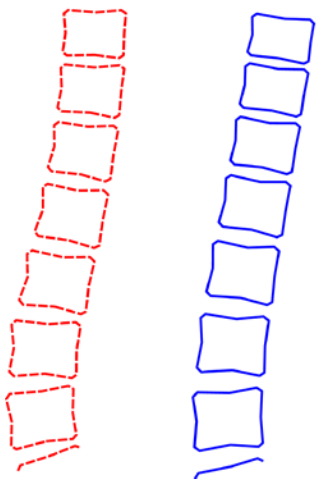

**5**<br>**(2.1%)**	Vertebral rotation at T10, L3 and L5 with resulting minor variations of lordosis and kyphosis and varying L4/L5 disc space.<br><br>Negative scores:<br>• Thoracic section tending towards a kyphosis but a flatter lordosis (anti-clockwise rotation at T10 and L3, clockwise rotation at L5).<br>• Smaller L5 anterior tilt and narrower L4/L5 disc space.<br><br>Positive scores:<br>• straighter T10-L2 section with a slightly greater lordosis from L3-L5 (clockwise rotation at T10 and L3, anti-clockwise rotation at L5).<br>• Greater L5 anterior tilt and wider L4/L5 disc space.	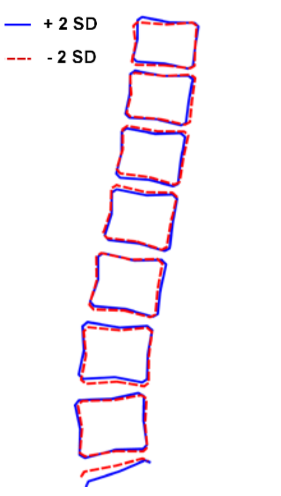	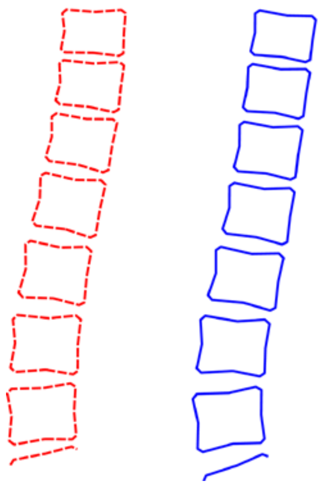
**6**<br>**(1.5%)**	Difference in anteroposterior vertebral body diameter cranially to caudally.<br><br>Negative scores:<br>• Smaller a-p diameters cranially; smaller than average at T10, T11 but wider than average at L3-L5.<br>• Smaller L4/L5 disc space.<br><br>Positive scores:<br>• More uniform a-p diameters; greater than average at T10, T11 but smaller than average at L3-L5.<br>• Greater L4/L5 disc space.	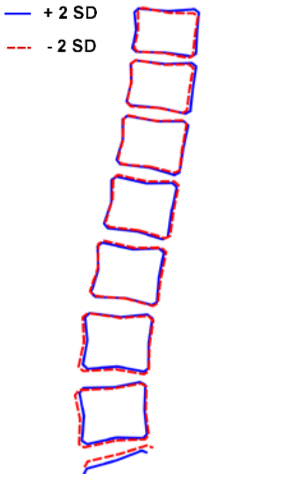	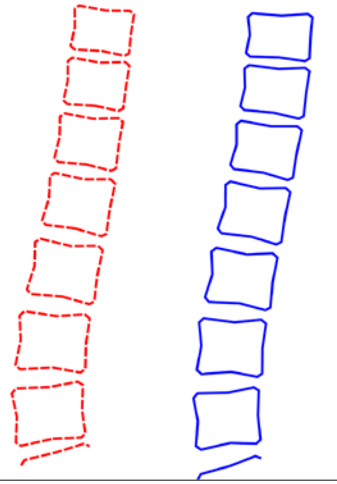

**7**<br>**(1.3%)**	Minor variation in a-p diameter at T10-T12 and at L4-L5<br><br>Negative scores:<br>• Smaller T10-T12, but larger than average L4-L5 a-p diameter. Squarer vertebral bodies in thoracic section.<br><br>Positive scores:<br>• Greater T10-T12 but smaller than average L4-L5 a-p diameter.	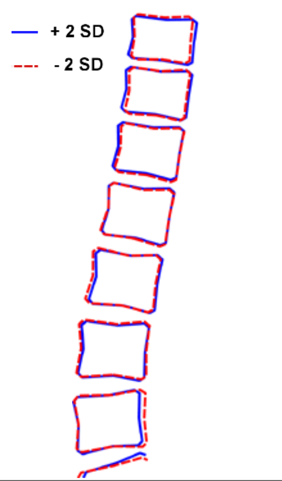	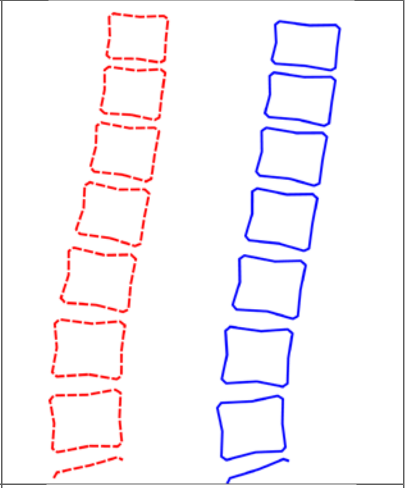
**8**<br>**(1.2%)**	Variation in L2-L4 vertebral body height, with consequent variation in disc space.<br><br>Negative scores:<br>- Smaller vertebral body heights, relatively larger disc spaces.<br><br>Positive scores:<br>- Taller vertebral body heights, relatively smaller disc spaces.	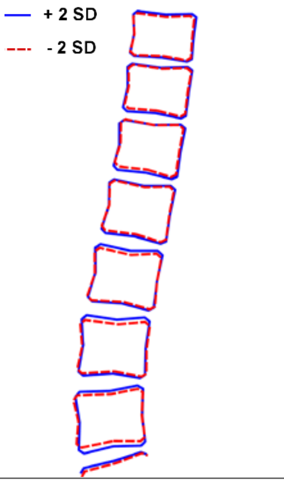	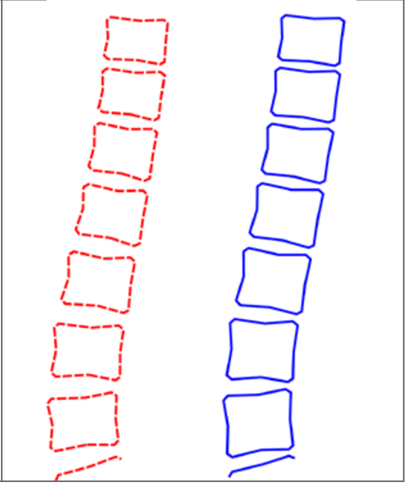

Figure S4. A description of the features varying in a coordinated fashion as identified by the spine mode scores SM1-SM8. The average score for each mode of the whole cohort is zero and positive and negative scores are described relative to the average. The percentage variation is the variance described by each principal component.
